# Drug Use among Street Children in Tehran, Iran: A Qualitative Study

**DOI:** 10.3389/fpubh.2015.00279

**Published:** 2015-12-23

**Authors:** Masoumeh Dejman, Meroe Vameghi, Payam Roshanfekr, Fatemeh Dejman, Hassan Rafiey, Ameneh Setareh Forouzan, Shervin Assari, Judith Bass, Renee M. Johnson

**Affiliations:** ^1^Department of Mental Health, Bloomberg School of Public Health, Johns Hopkins University, Baltimore, MD, USA; ^2^Social Determinants of Health Research Center, University of Social Welfare and Rehabilitation Sciences, Tehran, Iran; ^3^Social Welfare Management Research Center, University of Social Welfare and Rehabilitation Sciences, Tehran, Iran; ^4^Department of Psychiatry, University of Michigan, Ann Arbor, MI, USA; ^5^Center for Research on Ethnicity, Culture and Health, School of Public Health, University of Michigan, Ann Arbor, Michigan, USA

**Keywords:** qualitative, LMIC, street children, drug use

## Abstract

**Introduction and objective:**

Globally, children who work and live on the streets are at higher risk of undesired behavioral health outcomes, including increased drug use and abuse. Considering the rapid growth of this population in Iran and the lack of program planning that is partly due to a scarcity of research-based information, this study was conducted in 2013 to investigate drug use among street children in Tehran.

**^1^Method:**

With a qualitative design, we conducted a Rapid Assessment and Response Survey of street children in Tehran, 2012–2013. Data were also obtained from 10 focus group discussions with street children using semi-structured questionnaires and 27 in-depth interviews with key informants in governmental, non-governmental, and international organizations.

**Results:**

The variation in age at first use, type of drugs, and pattern of drug use were found based on ethnicity. Consuming of Alcohol was reported to be more among an ethnic group. Drug use problems were commonly described among families of street children. Children whose parents had drug-use problems described using drugs earlier than other children. Informants reported that families with drug-related problems used children for procurement of drugs. Children themselves described using drugs to cope with stress, and to reduce physical and psychological stressors and problems, such as fatigue, sadness, and pressure, resulting from frequent failures in life.

**Conclusion:**

These results suggest that intervention and prevention programs dealing with drug use of street children in Iran should include family and peers when addressing drug use by street children.

## Introduction

The phenomenon of street children is a major concern of the international community (1). Due to its hidden and isolated nature, it is difficult to estimate the exact prevalence of street children; however, the number appears to be tens of millions of children worldwide, which is probably on rise due to the rapid growth of the global population and urbanization (2).

In 2003, UNICEF classified street children into two general groups: (1) children who work on the streets to earn a living but are in contact with their families and have a sense of belonging to a family, although this degree of contact varies from once every few months to just about every day. UNICEF refers to these children as *“children on the street.”* (2) Children who search the streets for food and shelter and may consider other street people as their families. These children often have no family or are completely detached from a family. The United Nations Children’s Fund refers to these children as *“children of the street”* (3). There is one common component in all the definitions of the phenomenon of street children: all these children spend the major fraction of their time on the streets.

Different studies report a variety of causal factors associated with becoming a street child: socioeconomic factors and poverty (4–7), familial factors (6) such as low parental income and education, familial disruption, family size, decrease of family role in problem-solving for children, especially in urban areas, natural disasters, urbanization (6) dropping out of school, unplanned migration, wars (6), peer pressure, and the role of friends in persuading children to leave home and live on the streets (8).

There are no official statistics on the prevalence of street children in Iran, but it was unofficially estimated that 20,000 street children were living in Tehran, the capital city (9). Most of the children belong to low-income families of different age groups; many of them are immigrants and work in the streets of big cities, in streets with high volumes of traffic (10). It is estimated that 90% of street children are boys; the majority are over 12 years of age; almost 80% are illiterate or have had less than 8 years’ education, and at least one-quarter are Afghan. Nearly 80% of the children had a connection with their families (8).

Working and living on the streets cause countless dangerous consequences and place children at high risk for a range of poor outcomes, including drug use and HIV infection (11, 12). The risk of HIV infection among street children may be especially high due to marginalized social and economic situations, as well as substance use and other high-risk behaviors (13). In Egypt and Nigeria, drug abuse among street children was reported to be 62%, which increased the risk of other diseases (14).

Several studies have been conducted in Iran on health issues of street children (2, 15–22); yet, only a handful has addressed the prevalence of alcohol and drug use among this group of children. A study on working children in Tehran found that 40.1% reported alcohol consumption and 19.6% of the children reported drug use, which is much higher than in the general population for drug use (<0.1%) (23).

The present qualitative study was conducted to explore drug use behaviors of street children from the point of view of key informants and street children. This paper is part of a larger study of the rapid assessment and response (RAR) investigation of high-risk behaviors that seeks to present the status of drug use among street children; some of its results have been published before (24, 25).

## Materials and Methods

This qualitative study was conducted as part of the main study “The assessment of the demographics, work and high-risk behavior conditions of street children” conducted in Tehran in 2012–2013 using the RAR method (25, 26). This method is useful for obtaining information required for the design and development of health interventions and programs, and focuses on the characteristics of health problems, affected populations, key contexts and situations, health and risk behaviors, and social consequences. This method identifies the resources and opportunities for intervention, and helps with the design, preparation, and implementation of the interventions and programs (26).

The RAR method includes nine stages that include qualitative and quantitative methods (24, 25): formation of research teams, initial consultation, preparation of the studied region’s profile, field assessment, population and environment assessment, health problems assessment, health and risk behaviors assessment, assessment of current interventions, and development of the action plan. This paper focuses on the results of the qualitative interviews with street children and key informants as part of the health and risk behaviors assessment.

### Participants

The study population consisted of street children across Tehran and key informants working with the street children. The term “street children” included Iranian and non-Iranian children between the ages of 10 and 18 who spent a significant fraction of their day working or living on the streets of Tehran. In addition to the street children, key informants were interviewed. The key informants included experts and directors of governmental and non-governmental organizations providing services to street children in Tehran and international children’s organizations stationed in Iran (*N* = 27). The qualitative sampling was performed using the purposive and convenient method (described below).

### Data Collection

In the present study, we used two techniques for gathering data: individual interviews with key informants and group discussions with the street children.

#### Focus Group Discussion With Street Children

We used focus group discussions (FGDs) techniques to interview children because it is considered an ideal method for exploring people’s opinions and experiences connected with specific topics within a given cultural context (27, 28), and also children might feel more at ease than in an individual interview.

Ten group discussions were held with street children (average 5–10 children per group), with the children grouped by nationality (Iranian and non-Iranians). To obtain homogenous groups and enhance group dynamics (29), the groups were stratified by age (10- to 14-year-olds and 15- to 18-year-olds) and sex (boys and girls) (Table 1). Each group included both children who used services provided by non-governmental centers and children who did not use the services of these centers. Children were purposively (30) based on stratification recruited by participants with the assistance of peers from different parts of the city in cooperation with non-governmental organizations for street children. We used self-definition of participants to identify their ethnic background. Ten people were invited for each focus group (from the 120 children who were initially invited) to ensure a minimum participation rate of five people per focus group; 64 actually participated in the discussions. The most important reason for not participating was busy at work and lack of time.

**Table 1:** **Demographic characteristics of street children participating in group discussions**.

Groups	Girls		Boys	
Participants in each group	10–14	15–18	10–14	15–18
Nationality				
Iranian gypsy	5	6	5	5
Iranian non-gypsy	–	10	–	8
Afghan	6	8	5	6

Each interview was conducted by two members of the research team (the first, second, fifth, and sixth authors) as a moderator and observer, using semi-structured guide questions. The moderator started the interviews by presenting the aim of the group discussion. Participants were informed about confidentiality that participation was voluntary, and were informed of their right to withdraw from the study at any time during the focus group. The nature and purpose of the study were explained to each participant before his/her consent, which was confirmed by verbal consent. Then, the participants completed the first part of the guide questions (age, education, ethnicity, birth place, current job) that was attached to the consent form. If there were any questions, the moderators and observers responded individually. Permission to audiotape the interview session was sought orally prior to the interviews. Interviews were held in a quiet, convenient room in a non-governmental organization’s facility for street children.

The discussion started with asking participants regarding the characteristics of street children from their point of view, including asking how they define characteristics of different groups of street children such as age group, educational level, living and working places, and family characteristics such as type of and income of family, and family relationship. They were encouraged to talk openly about their knowledge and experiences about each issue. The moderator then asked about high-risk behaviors among street children such as patterns of drug use among street children, and probed for type of drugs, gender, age, and nationality differences, the place where the children use drugs, factors related to drug use, and sexual behaviors among street children from the children’s points of view (findings of the first part of questions and sexual behavior are presented in different papers). Probes were used to confirm concepts mentioned and to explore areas that the participants did not volunteer, especially regarding the factors related to drug use. The observer observed the atmosphere and interpersonal interaction in the focus group. Each focus group lasted 1.5–2 h and ended when no new issues seemed to arise. All of children got a sport t-shirt as a gift of participating in the study. The moderators and observers took field notes and discussed these immediately after each interview.

#### Individual Interviews with Key Informants

Twenty-seven individual interviews were also conducted with key informants from the following seven non-governmental organizations: the State Welfare Organization, Tehran Municipality, the Ministry of Health and Medical Education, the United Nations Children’s Fund in Tehran, and the UN Office on Drugs and Crime. Each interview was conducted by one interviewer in the key informants’ office. The guide question was similar to what was used in interviews of street children. Each interview lasted about 1.5 h. All interviews were recorded after getting permission and transcribed in Persian.

### Data Analysis

Data obtained from the interviews and group discussions were analyzed using the deductive and inductive content analysis method (31). The transcripts were read to derive “meaning units” (covering words, phrases, or paragraphs) using interview question topics as a guide, and were modified as information emerged from the narratives. Then, all data were reviewed for content for generating new codes or categories or modifying the categories and subcategories developed by induction. No new categories were found inductively from the data, and all codes and categories have been covered by interview topics for health and risk behavior assessment related to drug use.

Analysis started after each focus group and the interviews of key informants. The focus group struck a balance between looking at the big picture provided by the group as a whole and recognizing the operation of individual “voices” within each group (27). Due to the nature of the focus groups in which participants spoke freely, comments could not always be linked to a specific participant. Instead, extracted content was organized by recurrent codes relevant to study interests.

To enhance credibility, we selected participants from different ethnic groups, gender, and age groups. To assess dependability, peer checking by another team member to re-analyze some of the data was performed. There was more consistency for coding the naming categories; just the name of the second categories changed from factors related to drug use to reasons for drug use groups, and subcategories of type and methods of using drugs were merged to the main category of kind of drug use, looking across ethnic groups.

### Ethical Considerations

Given the legal obligations of the State Welfare Organization – as the main Guardian of street children – the Secretary of the State Welfare Organization’s Committee on the Prevention and Control of HIV issued permits for the implementation of the study and for interviewing the children. In each interviews with children and key informants, the interviewers explained the objectives of the study very clearly and stressed the optional nature of participation in the study. The children then gave verbal consent for participation in group discussions and were assured of confidentiality and informed of their right to withdraw from the study at any time during the interview and were free to decide whether to participate in the research. Permission to audiotape the interview was asked orally from the participants prior to the interviews. To establish rapport, the children were asked to take part in the discussions under any nicknames they wanted and to refrain from revealing their family names.

## Results

Twenty-seven key informants participated in the study – 16 non-governmental, 9 governmental, and 2 international experts – along with 64 street children. The demographic details of 13 street children discussion groups are presented in Table 1.

The participants’ views were analyzed within the following three categories: type of drug used, looking across ethnic groups, reasons for drug use, and drug use problems. The three categories are presented in detail below.

### Type of Drug Use, Looking Across Ethnic Groups

Most of the key informants believed that more than 80% of the working and street children use drugs. They believed that the use of drugs, such as crystal, opium, cannabis, and pills, and alcohol consumption are very common among working and street children and tend to start at younger ages, often around age of 10 or 11. In addition, they believed that the use of drugs, especially crystal, and even alcohol use, are associated with aggressive behaviors, increased libido, and reduced sexual inhibition. In the discussion groups with children, they also reported being familiar with addiction and drug use, and reported often witnessing drugs such as crystal and injection drugs being used in parks and on the streets. They talked about the involvement of some children in procuring drugs for their family members, or for other addicts, or sometimes even suggestions by vendors to sell drugs themselves.

In the experience of four NGO experts, drug use and addiction are increasing among different ethnic group’s families who immigrated to Tehran and the children of these families are also involved in drug use and drug dealing.

In group discussions with Iranian boys (aged 14- to 18-year-old), they reported that children start with smoking hookah and cigarettes and gradually turn to stronger drugs, such as Naas, opium, opium extract, crystal, heroin, ecstasy pills, and alcohol. They know the existing varieties of drugs and also know how to use them. Both Iranian boys and girls (in both age groups) also pointed out children using drugs in parks, and stated that children and adults use drugs on the streets side by side. The most widely used drugs in the neighborhood of Iranian girls aged 14–18 years were reported crystal and hashish, hash cigarettes, or hash in foil.

They smoke pipe too. Its round with a hole, just like a pen. They put the stuff in it and stick a lighter underneath to heat it up. Some smoke fruit juice with pipe, and call it a cocktail. My aunt smokes this. They pour crystal in a pipe, pass a wire through it, and then pass it through that hole, and that is how they smoke crystal [group discussion with Iranian boys (14- to18-year-old)].

According to key informants, both Gypsy boys and girls drink alcohol; although gypsy girls’ consumption of alcohol to be less than their drug use. Yet, Gypsy girls (10- to 14-year-old), were also reported that some of them did indeed drink alcohol themselves.

Addiction is much higher among our [Gypsy] girls because girls don’t drink alcohol as much, so they turn to drugs. They’re not too much into alcohol. Maybe when their father and cousins are drinking, the girls go drink what’s left of their shots. But generally, girls don’t drink. It’s interesting that Gypsies consider it bad for women and girls to drink with the men.

I have drunk a hundred times. I was angry. I had argued with some girl. She said, do you want this, and I said no. I went home. I saw a bottle. Thought it was water. I gulped it down. It was so bitter, so bitter. I beat up my father [Gypsy girls (10- to 14-year-old groups)]

(NGO expert)

Drug use was also relatively commonplace among Gypsy children, though according to one key informant, opium and injection-drug use are mostly limited to adults. According to most 10 of the key informants who worked with Gypsy children, the most common drugs used by these children were crystal meth and crack, which were cheaper than other drugs. This was consistent with results of the discussion with gypsy girls (10- to 14-year-old) who mentioned that they were quite familiar with crystal and knew how to use it and Gypsy boys from the younger discussion group (10- to 14-year-old) who also knew their way around drugs and knew how to use crystal, hashish, and heroin.

It had gotten so bad, and I took in a lot of smoke. I was 14. My mother had gone out and my father was in prison. I picked up my mother’s apparatus, I didn’t know what it was, and then I smoked it, I smoked just because. Then it got me high, so I smoked again. I got it refilled and smoked like a chimney. Then I began to feel unwell. I went outside to sweep the yard. Suddenly I felt dizzy and threw up and then I fell unconscious for two whole days [Gypsy girls (14- to 18-year-old group)].

According to key informants, it appears that Afghan children are less involved with drug use compared to other children.

Compared to other children, addiction rates are very low among Afghan children and their families. Afghan families have very much preserved their family unit. They tend to protect and control their families, and family principles are important to them. I believe Afghans are the most conservative, and have clear relationships and responsibilities; they control and supervise, and are less likely to get involved in risks and danger.

(NGO expert)

In the group discussions, the younger and older Afghan boys reported being familiar with drugs, such as crystal and injection drugs, and the older boys believed that drug use was far less prevalent among Afghan children than among Iranian children, and that those Afghan children who did use drugs had learned the behavior in Iran and Pakistan. They reported that Afghans had immigrated to work and earn money and were frightened of using drugs and would not involve themselves in drug use behaviors unless they were deceived or wanted to copy such behaviors from Iranian children.

### Reasons for Drug Use

The reasons for drug use were divided into two categories: family-related reasons and reduction of mental pressure.

#### Family-Related Reasons

According to key informants and the group discussions with street children, addiction is common among the families of street children. They reported that addiction was more common among the men of the family, that is, the fathers and brothers, but in many cases, mothers also used drugs. The key informants believed ethnic characteristics to be highly influential in the families’ degree and type of involvement with drugs. Most experts believed that, among Gypsy families, both men and women are abundant drug and alcohol users.

Key informants also reported that children from families with addiction tended to become involved with drug use earlier. In addition, they reported that street children turn to drugs to reduce their sadness and the pressures of their numerous failures in life. But, one of the children, in group discussions, pointed to the sorrow from the breakdown of emotional relationships as a reason for using drugs.

“There was once even a 6-year-old girl who used drugs by herself, since both her parents were addicts, she had learnt their ways and would sit and smoke crack.”

(NGO Official)

#### Reduction of Psychological Stress

Some of the children in the group discussions especially among Gypsy and Iranian groups reported that they used drugs to reduce their physical and mental pressures, such as fatigue, sadness, sorrow, and stress, caused by various problems. They considered their parents’ pressures on them for finding a job and earning a living a major stress factor.

Group discussion with 10- to 14-year-old Gypsy girls: Her father bugged her. Since her parents bugged her so much, and told her they would not give her any pipes to sell if she did not bring home (2000 RLs) less that 2 $ every day. They said they wouldn’t let her marry or go out with her fiancé.

According to a key informant, exhaustion from work can also be a reason for gypsy girls’ turning to drugs.

I thought about this problem myself. Perhaps too much exhaustion and hard work, since girls have to constantly work and since they get pregnant from a very young age, say, 17, and by the age of 20–21, they have like 3 children, and their body and face look haggard. But their husbands are only 21 years old, just now in their prime, becoming better-looking than before and thinking about having girlfriends. The damage to women and girls is huge.

(NGO Official)

According to key informants’ opinions, young children need protecting and preserving factor against addiction. But these children often do not experience attachment at all or else it is very faded. Even those living with their family, given the large number of children in the household, the parents cannot support them all. In addition, the despair and failures these kids experience for their basic needs like, food, good clothing, a home, being respected, etc.; they receive none of these services, and so a combination of all these factors leads the children to resort to drug abuse from all the pain and sorrow. It is also mentioned that boys need alcohol to help them play their social roles and reduce their anxiety.

Without getting drunk, they have very little self-confidence. If they want to fight, they have to get drunk; otherwise they won’t be able to fight. There was a boy who wanted to see me, and I went over and looked for him for a whole week to see what he wanted. At the end, when I found him, he was drunk, and he said that he could now tell me everything for he was drunk. They have to get drunk to dare go and talk to their own girlfriends for the first few times or have sex with them; otherwise they won’t dare even talk.

(GO Official)

##### Other Reasons

*Key informants mentioned that* street children have a higher daily income than their peers, so they – particularly younger children – are targeted by addicts who intend to take advantage of their income for their own good by involving them in drug use. Additionally, children in the 14–18 age group also mentioned that street children took drugs as a pain-killer or for decreasing the stress, and considered competition with peers. Peer pressure was considered among the main causes of turning to drug use.

Iranian boys’ group discussion (aged 14–18 group): Right now, 9–10-year-old kids are also addicted. For example, their kids are given opium for the pain in their legs, or when they have a toothache, they put opium extract on the tooth. Even if their mothers don’t give them these drugs, their friends do.

### Drug-Use-Related Problem

One of the important drug-use-related problems with street children was getting involved in drug dealing. Some informants reported that, in families with addiction, the adults tend to take advantage of the children for supplying their drugs; it also occurred because of the pressure from the family for procuring drugs. An NGO informant also reported that exhaustion from hard work and low income of the street children toward higher-income trades like drug-pushing when they reach adolescence.

We had this one kid who was very smart. He was also very tiny. He was in the drugs business. He said he went to get drugs for his father from XXX street, and for his mother too, for the pain in her leg. When we went to his home, we saw that it was a place for drug use. He was sent to get drugs because he was young. Everyone, his father, his mother and his older brothers, were all addicts.

Group discussion with 10- to 14-year-old Afghan girls: Their father is addicted, so he sends his child to buy him drugs. I used to do this myself. My father would send me out to buy drugs. I used to cry, because I didn’t want to go, but my father would have beaten me up if I hadn’t gone.

(NGO Official)

A number of informants (*N* = 7) pointed to children’s drug dealing along with their street vending for earning an income, which might be an act of willingness or might be under the family’s pressures. The children mentioned that, sometimes, addicts or drug pushers suggest they sell drugs instead of selling fortune cards, which does not really pay off well, and so sometimes they agree to it.

Group discussion with 14- to 18-year-old gypsy girls: I used to sell alcohol and crystal, by the kilo. I would get it from the kids and sell it. I mean, it only took 10 min for me to run out. There was a lot of demand, so I got rid of them all. I was very nifty.

Family addiction itself was considered the source of many other problems in the families of these children: the head of the family losing his job, spending all income from work and from selling family properties for drug supply, imprisonment, and parents’ aggression toward one another and toward the children, and the parents’ separation or divorce. Street children and informants considered that the children of addicted mothers were at risk due to their mother’s lack of attention and inadequate care.

## Discussion

Results of the study showed that perceptions of key informants and street children collectively suggest that drug use among street children varies across different ethnic groups in terms of type of drug used, age of use, and gender. Informants and street children described that drug and alcohol use is higher among younger (10- to 14-year-old) and older (15- to 18-year-old) Iranian and Gypsy boys and girls compared to Afghan boys and girls. According to informants who had worked with Gypsy children, crystal and crack are the most common drugs used by them, as they are more affordable for street youth. From the discussion groups with Gypsy boys and girls, there were reports that both genders consume alcohol. The most common drugs used by 14- to 18-year-old Iranian girls were reported to be crystal and cannabis rolled up in foil and in cigarettes, and the most common drugs used by Iranian boys were reported to be Naas, opium and opium extract, crystal, heroin, ecstasy pills, and alcohol.

Our findings – based on key informants and street children’s opinions – reported no differences in drug use among boys and girls except in one group (Afghan Group). Whereas, a household survey conducted in Kerman, Iran, with a group of 10- to 12-year-old children showed drug use to be associated with gender and to be more prevalent among boys, which is different from what we found (32). This difference might be attributable to the geographic and urban differences between Kerman and Tehran, the differences in the study sample characteristics, or to the study methods – our study used open-ended qualitative questions, which allowed the children to answer the questions and talk openly.

According to results of the present study, many children reported having family (mainly a father) who had drug use problems, and based on key informants’ opinion, children from families with addiction tended to become involved with drug use earlier. Studies show that the families of street children can play both positive and negative roles in initiating drug use and in facilitating its quitting. A cohort study conducted by Khademi (33) among 50,000 adults in Iran showed that children commonly expressed that they picked up their information about drugs from their friends (62%), family/guardian (16%), and teachers (8%). In a study conducted in 2009 by Hudson et al., many of the young generation had begun drug use from early ages due to their parents’ drug use. Moreover, according to the literature, illicit drug use in parents is highly associated with substance use in adolescence (34–37). Embleton also revealed a direct correlation between drug use and alcohol consumption among family members and drug use among street children. The role of family is also important as social support. The lack of family care in children who have left home and who now share a place to sleep with their friends or else sleep alone on the streets or in support centers or their workplace, living in publicly rented shelters – all increase the likelihood of drug use (11). So, implementing intervention programs actively involving families on risk assessments and risk reduction could be more effective in decreasing high-risk behaviors among street children. Results of a study conducted in 2012 by Milburn et al. (38) showed that 12-month interventions aimed at reuniting adolescents who have run away from their home due to family conflicts were significantly effective in reducing the risk of alcohol consumption and resort to heavier drugs’ use, criminal behavior, and sexual risks. The perceptual framework containing interventions emphasizes the importance of creating a positive family atmosphere, valuing one another, improving the family function through finding two-way solutions to conflicts, learning to recognize and effectively manage emotions, and learning and practicing problem-solving skills.

The results of the present study show that street children know how to use drugs, have experienced addiction from an early age, and were influenced by their friends on how to use drugs. A quantitative study was conducted by Shoghli et al. (39) In Iran, Shoghli and Mohraz also found a significant difference in terms of drug use between street children based on drug use problem of friends and families. The study of Emlenton on 146 street children in Kenya (40) showed that friends play a dual role in street children’s drug use and quitting patterns. According to results of their study, the majority of street children (84%) sniffed glue to copy their friends. Most drug users were first introduced to drugs through their friends (71%). According to results of a controlled confounding factors study conducted in 2010 by Latimer et al., adolescents who had drug-using friends were twice as exposed to alcoholism and 2.5 times more exposed to use drugs other than alcohol compared to adolescents without addicted friends (33). Although some friends have a negative role in encouraging drug use, other friends are able to play a protective and informative role in encouraging quitting. These all support the unique role of peers who are more familiar with the culture and behavior of their own ethnic groups on education of reduction of high-risk behaviors, such as drug use among children – especially street children (41).

In our study, one of the reasons of using drug by street children was deceiving by their friends to the belief that drug use may help them with sleep and reduce their stress that they had been. The children also explained that older street children and adolescents, drug pushers, and the street children’s group leaders forced them to buy drugs even if they did not want to. These findings are consistent with other studies (42–45). In a quantitative study, the highest frequency of reasons for street children’s drug use was reported to be curiosity, pleasure, fun, peer pressure, and reduced mental pressure and pain, in descending order of frequency (46). Another study in Iran also showed that the main reasons for drug use by street children were forgetting problems (32%), feeling warmer (24%), feeling better (17%), and getting used to drug use (14%) (47).This results also consistent with the report of other countries. The street children in India explained that using drugs reduced their hunger, helped them sleep better, reduced their stress, helped them forget the past, and made them more daring and more powerful to fight for finding food and survival (45). Low self-esteem or self-confidence, boredom, and escape from the reality of homelessness and unemployment were among the other motivations for drug use that, according to the children, is a way of killing time (38). So, including self-efficacy program and involving street children in social activities beside their daily life is recommended to be included in any intervention programs for these children.

Results of the present study demonstrate the vital role of families and peers in drug abuse and alcohol consumption by Iranian street children, which supports Bronfenbrenner’s socio-ecological model of human development, one of the most widely, used theoretical frameworks in the area of drug use. Based on this theory, drug use behaviors of street children is under the influence of their family and peers (32). Achieving better results through drug use prevention programs and interventions targeting street children requires the integration of intervention programs targeting their families and peers. To prevent children from leaving home, serious consideration should be given to providing social support for street children and their families appropriate with the culture of different ethnic groups, providing accessible health care and counseling services, and prevention of violence using non-violent methods such as education and communication for their family.

### Limitation

The present study illustrates the opinions of the key informants and street children by the focus groups and individual interviews and should for methodological reasons not be generalized to other contexts. The group of participants with 10- to 14-year-old aged had some difficulty and shyness in talking about the high-risk behaviors, and it was so difficult to keep them not to talk with others during the discussion. FGD differs from individual interviews in that, in addition to the reactions of the leader, all group members influence how the discussion proceeds and the participants may attempt to provide a “desirable” response. Regarding the selection of participants, recruiting of participants often involves an increased dependency on gate-keepers who were peers from different NGOs of street children. They may screen potential participants. Alternatively, these people might be more accessible in the city.

## Author Contributions

MD: substantial contributions to the conception or design of the work, interpretation of data for the work, drafting the work or revising it critically for important intellectual content, final approval of the version to be published, agreement to be accountable for all aspects of the work in ensuring that questions related to the accuracy or integrity of any part of the work are appropriately investigated and resolved. MV, HR, PR, FD, and ASF: substantial contributions to the conception or design of the work, drafting the work for important intellectual content, final approval of the version to be published, agreement to be accountable for all aspects of the work in ensuring that questions related to the accuracy or integrity of any part of the work are appropriately investigated and resolved. SA, RJ, and JB: Interpretation of data for the work, revising it critically for important intellectual content, final approval of the version to be published, agreement to be accountable for all aspects of the work in ensuring that questions related to the accuracy or integrity of any part of the work are appropriately investigated and resolved.

## Conflict of Interest Statement

The authors declare that the research was conducted in the absence of any commercial or financial relationships that could be construed as a potential conflict of interest.
